# Trait aspects of auditory mismatch negativity predict response to auditory training in individuals with early illness schizophrenia

**DOI:** 10.1186/s40810-017-0024-9

**Published:** 2017-06-09

**Authors:** Bruno Biagianti, Brian J. Roach, Melissa Fisher, Rachel Loewy, Judith M. Ford, Sophia Vinogradov, Daniel H. Mathalon

**Affiliations:** 1 Department of Psychiatry, University of California at San Francisco, San Francisco, CA, USA; 2 Department of Health Sciences, University of Milan, Milan, Italy; 3 Department of Mental Health, San Francisco VA Medical Center, San Francisco, CA, USA; 4 Department of Psychiatry, University of Minnesota, Minneapolis, MN, USA

**Keywords:** Schizophrenia, Cognitive training, Neuroplasticity, Mismatch negativity, Biomarkers

## Abstract

**Background:**

Individuals with schizophrenia have heterogeneous impairments of the auditory processing system that likely mediate differences in the cognitive gains induced by auditory training (AT). Mismatch negativity (MMN) is an event-related potential component reflecting auditory echoic memory, and its amplitude reduction in schizophrenia has been linked to cognitive deficits. Therefore, MMN may predict response to AT and identify individuals with schizophrenia who have the most to gain from AT. Furthermore, to the extent that AT strengthens auditory deviance processing, MMN may also serve as a readout of the underlying changes in the auditory system induced by AT.

**Methods:**

Fifty-six individuals early in the course of a schizophrenia-spectrum illness (ESZ) were randomly assigned to 40 h of AT or Computer Games (CG). Cognitive assessments and EEG recordings during a multi-deviant MMN paradigm were obtained before and after AT and CG. Changes in these measures were compared between the treatment groups. Baseline and trait-like MMN data were evaluated as predictors of treatment response. MMN data collected with the same paradigm from a sample of Healthy Controls (HC; *n* = 105) were compared to baseline MMN data from the ESZ group.

**Results:**

Compared to HC, ESZ individuals showed significant MMN reductions at baseline (*p* = .003). Reduced Double-Deviant MMN was associated with greater general cognitive impairment in ESZ individuals (*p* = .020). Neither ESZ intervention group showed significant change in MMN. We found high correlations in all MMN deviant types (rs = .59–.68, all ps < .001) between baseline and post-intervention amplitudes irrespective of treatment group, suggesting trait-like stability of the MMN signal. Greater deficits in trait-like Double-Deviant MMN predicted greater cognitive improvements in the AT group (*p* = .02), but not in the CG group.

**Conclusions:**

In this sample of ESZ individuals, AT had no effect on auditory deviance processing as assessed by MMN. In ESZ individuals, baseline MMN was significantly reduced relative to HCs, and associated with global cognitive impairment. MMN did not show changes after AT and exhibited trait-like stability. Greater deficits in the trait aspects of Double-Deviant MMN predicted greater gains in global cognition in response to AT, suggesting that MMN may identify individuals who stand to gain the most from AT.

**Trial registration:**

NCT00694889. Registered 1 August 2007.

## Background

Dysfunction in the coordination of neural activity during auditory processing is well-documented in individuals with schizophrenia [1, 2]. Impairments in basic sensory processing abilities, including encoding and decoding of information, are present early in the course of the illness and in individuals at risk for schizophrenia [3, 4]. An established body of evidence suggests that impaired early processing operations in the auditory system are associated with disturbances in fronto-temporal language processing networks that in turn contribute to widespread aberrant neurocognitive-perceptual processes, including deficits in verbal encoding, working memory, and episodic and semantic memory [5, 6]. These cognitive impairments have been observed across the illness course of schizophrenia, are present in antipsychotic-naïve first-episode individuals and in individuals at risk for psychosis [7, 8], and predict the transition from prodromal to first-episode psychosis [9].

Training of auditory processing has evolved as a target in the development of interventions for the cognitive impairments characteristic of schizophrenia. The computerized auditory training (AT) program we have studied simultaneously targets feedback and feed-forward operations in the auditory system [10]. The training exercises target feed-forward auditory perceptual processes by placing implicit, increasing demands on discrimination of basic auditory and verbal stimuli. Feedback attention and cognitive control operations are engaged by signalling correct/incorrect trials and by embedding the psychophysical training within increasingly complex auditory and verbal working memory/verbal learning trials. The mechanism of action is thus posited to be the “re-tuning” of the bi-directional operations between temporally detailed resolution of auditory inputs in auditory cortex, prefrontally-mediated attention, and auditory/ verbal memory functions. Indeed, emerging electro- and magneto-encephalographic data indicate that AT enhances both early representations in primary auditory cortex and auditory sensory gating [11, 12], as well as both early and later task-related activity in prefrontal regions [13]. Improved efficiency in distributed prefrontal-temporal auditory systems is therefore thought to drive improvements in untrained higher-level cognitive operations [14, 15].

Two randomized clinical trials (RCT) that investigated the effectiveness of AT in schizophrenia reported improvements in general cognitive performance, with moderate to large effect sizes observed in individuals with recent-onset (d = 0.56) and chronic (d = .86) schizophrenia-related disorders [16, 17]. Despite these promising results, two recent studies found overall improvement on the AT exercises but no transfer of these gains to other cognitive outcome measures [18, 19]. Additionally, in the two RCTs from our group, while we observed significant improvements in general cognition in the participants who received AT relative to the participants assigned to the computer game control condition [16, 17], ~40% of the participants did not show gains beyond expected practice effects [20, 21]. Furthermore, participants who showed greater improvements in the auditory processing speed metrics embedded in the training program showed larger gains in general cognition, consistent with the idea that improved auditory system processing could translate to enhanced cognitive performance [16, 17].

Why do some individuals with schizophrenia show evidence of robust cognitive improvement, while others do not? The observed variability in cognitive gains could be due to the heterogeneity of impairment of the prefrontal-temporal neural systems that underlie auditory processing. Investigating the neurophysiological bases of auditory system dysfunction in schizophrenia could therefore be key to identify predictors of response to AT and guide mechanistically informed, personalized treatments. Among the various indexes of early auditory processing, mismatch negativity (MMN), an event-related potential (ERP) that is elicited pre-attentively when an infrequent deviant sound violates an established pattern of repeated standard sounds [22], has been proposed to be used to predict and track response to AT [23–26]. The MMN response is seen as a negative displacement in particular at the frontocentral and central scalp electrodes (relative to a mastoid or nose reference electrode) in the difference wave obtained by subtracting the ERP to frequent, “standard”, stimuli from that to deviant stimuli. Recent interpretations of the MMN suggest that the resolution of auditory signals permits the short-term formation of memory traces of the standard sounds that code predictions of future auditory events [27]. The MMN amplitude is thought to signal the prediction error that occurs during implicit perceptual learning when the auditory deviant violates the auditory expectancy [27–30]. Because the echoic memory system, like MMN, functions preattentively, deficits cannot be easily ascribed to impaired attention, emotion, or motivation. To date, MMN constitutes the most sensitive readout of automatic auditory deviance processing, and probably the unique measure for the neurophysiological correlates of echoic sensory memory [27].

Echoic sensory memory can be modulated by means of pharmacological agents, and these effects can be reliably assessed by changes in MMN. Because MMN depends critically on *N*-methyl-D-aspartate receptor (NMDAR) function [31], the modulation of NMDAR function with pharmacological agents alters the MMN: for example, the NMDA antagonists ketamine and PCP reduce the MMN in healthy participants [32, 33]. Furthermore, invasive intracortical recording studies in monkeys demonstrated that local infusion of competitive and noncompetitive NMDA antagonists block the generation of the MMN [34]. In addition, neuromodulatory transmitters of NMDAR function, including acetylcholine or serotonin, alter the MMN [35, 36].

The reduced MMN amplitude, well documented in chronic schizophrenia, first episode psychosis, and even individuals at risk for psychosis [37–40] is thus posited to reflect NMDAR-mediated compromised echoic memory formation and predictive coding [27, 29]. More broadly, many authors have proposed the abnormal regulation of NMDAR by neuromodulatory transmitters as the mechanism that underlies the aberrant functional integration among brain regions in schizophrenia (i.e., dysconnectivity) [41, 42].

Besides being consistently reduced across the course of illness, MMN has other important characteristics that support its role as a potential vulnerability marker for schizophrenia and its use as a repeated measure in intervention studies, including substantial heritability [43], lack of order or practice effects and good test-retest reliability (ranging from .3 to .7 in studies where individuals are tested on more than one occasion [44–46]), and trait-like stability over long (e.g., 12-month) retest intervals in both healthy participants and individuals with schizophrenia, with ICCs approaching 0.90 [47]. Additionally, MMN is shown to be associated with cognitive abilities and psychosocial functioning in normal participants [6] and in individuals with schizophrenia [37, 48, 49], and independent of fluctuations of clinical state and symptoms [47].

Despite a general convergence of research findings showing reduced MMN amplitude in response to a Frequency-Deviant [50] or a Duration-Deviant [51–55] stimulus, there is also a high degree of variability among studies, with some reporting normal MMN, especially in individuals with schizophrenia early in their illness and in those who are unmedicated [51, 54, 56–60]. One possible explanation for this heterogeneity is the variable degree of impairment of the fronto-temporal networks underlying auditory processing and signal resolution of auditory inputs. Arguably, individuals with the most severe auditory processing deficits may stand to gain the most from targeted auditory training. In this context, reduced MMN amplitude may identify the individuals who have the greatest potential to benefit from AT, including achievement of more general cognitive gains.

Another hypothesis for inconsistent reports of MMN amplitude reduction is based on the high degree of heterogeneity in the fundamental mechanisms of short term synaptic plasticity found in schizophrenia [61–68]. Although the neurobiological underpinnings of this heterogeneity are at present unknown, some authors have considered NMDAR hypofunction to be a possible mechanism underlying plasticity deficits [42]. In this context, MMN can be considered an index of experience-dependent short-term synaptic plasticity in the service of auditory sensory/perceptual learning [23, 69], the proximal mechanism that is targeted by AT. Normal MMN amplitude would indicate intact synaptic plasticity in the prefrontal-temporal neural systems that underlie auditory processing. This view of MMN as an index of synaptic plasticity is corroborated by previous findings showing that larger MMN amplitude predicts greater training-induced changes in cognitive functioning and language acquisition in non-clinical populations [24, 25]. Therefore, it is possible that impairments of the mechanisms of short-term plasticity in prefrontal-temporal networks hamper psychophysical learning during AT. If so, individuals with greater impairments in synaptic plasticity, as indexed by reduced MMN amplitude, might not be able to generate and sustain successful learning in response to the training trials, and this inability in turn would be manifested as lower or absent general cognitive gains after AT.

From a methodological point of view, a third possible explanation for inconsistent reports of reduced MMN amplitude in individuals with schizophrenia may lie in the magnitude of the psychophysical deviation and in the different types of deviant stimuli used to elicit MMN, since it appears that distinct neural populations process different dimensions of auditory deviance [70–72]. Given the potential for heterogeneity among individuals with schizophrenia in terms of which type of MMN is most affected by their particular variant of the illness, we studied both Frequency-Deviant and Duration-Deviant MMN, as well as a “Double-Deviant” MMN elicited in response to a single stimulus that combined Frequency and Duration deviance features. Combining deviance features in a single stimulus has previously been shown to enhance the amplitude of MMN in healthy participants [70–72], and is theorized to have greater sensitivity to schizophrenia than stimuli that are deviant in only a single feature [70–72].

Above and beyond its role as a predictor of treatment response, and because of its trait-like stability, MMN may serve as an indicator of neurophysiologic changes in the central auditory system resulting from AT. Studies conducted with healthy participants support this hypothesis, showing persistent MMN improvements after auditory discrimination training [73–75]. Similarly, studies of auditory perceptual learning in older adults found changes in sensory ERPs following training that predicted behavioral improvements in perceptual tasks and global cognitive gains [76, 77]. However, emerging evidence from studies conducted in schizophrenia seems to cast doubt on this hypothesis. Recently, one study tested the hypothesis that a brief two-week auditory training in individuals with schizophrenia would result in an increased MMN amplitude, and found significant improvements in verbal working memory [78], but no training-specific effects on MMN amplitude or latency, possibly because of the small sample size, narrow range of auditory stimuli, and/or an insufficient number of training sessions to drive neurophysiological changes [79]. Therefore, studying a larger group of individuals with schizophrenia undergoing a more intensive auditory training program previously shown to induce changes on indices of neurophysiological parameters [11–13] could provide important insights on the role of MMN as a proxy for the underlying changes in auditory processing induced by AT.

In this study, we assessed cognitive performance and auditory MMN in individuals with schizophrenia randomly assigned to intensive auditory training (AT) or computer games (CG) before and after the intervention. We enrolled participants with a schizophrenia-spectrum illness within 5 years of treatment initiation or illness onset. We hypothesized that individuals in AT but not in CG would have a non-zero relationship between baseline MMN and cognitive gains following treatment. Regarding the directionality of this relationship, our leading hypothesis was that individuals with greater auditory processing deficits, as indexed by reduced MMN amplitude, would show greater cognitive improvements after AT, but not after CG. The competing hypothesis was that normal MMN amplitude would reflect intact short term plasticity and therefore help identify the individuals who would show the greatest cognitive gains after AT, but not after CG. Finally, we hypothesized that individuals with schizophrenia would show increased MMN amplitude after AT but not after CG, and that MMN changes would correlate with cognitive changes in the AT group but not in the CG group.

## Methods

### Participants

Fifty-six participants early in the illness course of schizophrenia, schizophreniform, or schizoaffective disorder (ESZ) completed an auditory cognitive training treatment study protocol at the University of California, San Francisco (ClinicalTrials.gov NCT00694889) and completed MMN measures. This sample comprises the subset of participants from the total trial sample (which has been reported upon previously [17]) who agreed to participate in the EEG experiment. Participants met the following inclusion/exclusion criteria: (1) onset of first psychotic episode or initiation of antipsychotic medication within the past 5 years; (2) age 14–36 years; (3) fluent and proficient in English; (4) intelligence quotient (IQ) ≥ 70; (5) no neurological disorder; and (6) no DSM-IV substance dependence in the past year.

All ESZ participants had achieved outpatient status for at least 3 months, and participants taking antipsychotic medications (*n* = 52) were on a stable dose for at least one month prior to study participation. Four participants were not taking antipsychotic medications during the study. The study was approved by the Institutional Review Board of University of California, San Francisco. Adult participants and parents of minors provided written informed consent, and minors provided written assent.

Eligible ESZ participants completed a battery of clinical, neuropsychological and EEG assessments. Baseline assessments were conducted prior to randomization. ESZ participants were randomly assigned to auditory training (*n* = 27) or computer games (*n* = 29). ESZ participants were loaned laptop computers and participated in the intervention at home, except for 1 training subject who preferred to participate in the laboratory. ESZ participants were asked to participate for 40 h (1 h/d, 5 d/ wk., for 8 wk), followed by post-training assessments.

The auditory training (AT) program, provided by Posit Science Corporation, has been described in detail previously [10]. It consists of six computerized exercises designed to improve speed and accuracy of speech-related information processing and auditory working memory. ESZ participants rotated through 4 exercises each day of training, with training on more elemental auditory processing (frequency-modulated tone discrimination, temporally-modulated syllables processing) more heavily weighted during the first 20 h, and higher-level verbal and whole-language exercises emphasized during the second 20 h.

The computer games (CG) control condition controls for the effects of computer exposure, contact with research staff, monetary compensation, and non-specific engagement of attention, executive functions, and motivation. ESZ participants in the CG condition rotated through a series of 16 different commercially available games for the same number of hours as participants in the AT group, playing 4–5 games on any given day [17].

All ESZ participants received compensation for study participation, and payment was contingent on study participation and not performance (for details about the payment schedule, see Fisher et al. 2015). During the intervention, ESZ participants were free to receive treatments by clinicians who were not involved in the study (e.g. medication management, psychoeducation, psychotherapy).

### Clinical and cognitive assessments

All assessment staff were blind to group assignment. Eligibility diagnoses were determined using the Structured Clinical Interview for DSM-IV TR [80]. Symptoms were assessed with the Positive and Negative Syndrome Scale (PANSS) [81]. Clinical interviews were conducted by a trained research assistant or clinical psychologist. Cognitive assessment staff were trained on manualized assessment procedures by M.F. An abbreviated battery of Measurement and Treatment Research to Improve Cognition in Schizophrenia (MATRICS)-recommended measures was administered [21]. Raw scores were converted to *z-*scores using age-appropriate normative data provided in testing manuals and age-appropriate, published normative data. We used the Global Cognition score (average *z-*score across all MATRICS measures) as our primary cognitive outcome.

### MMN paradigm

We assessed three types of MMN using a two-Deviant paradigm and a Single-Deviant paradigm. The two-Deviant paradigm assessed Frequency-Deviant MMN and Duration-Deviant MMN. In this paradigm, 80% of the stimuli were standard tones (50 msec, 633 Hz), 10% were duration deviants (DUR: 100 msec, 633 Hz), and 10% were frequency deviants (FREQ: 50 msec, 1000 Hz). The Single-Deviant paradigm assessed the MMN elicited by a Frequency + Duration Double-Deviant (DBL) stimulus. In this paradigm, 90% of the stimuli were standard tones (50 msec, 633 Hz), and 10% were double-deviants (DBL: 100 msec, 1000 Hz). Across paradigms, all tones had 5 millisecond rise/fall times and were presented with a 500 msec stimulus onset asynchrony at 78 dB sound pressure level via Etymotic ER3-A insert earphones. The paradigms were administered in four separate blocks (two blocks of the two-Deviant paradigm, two blocks of the Double-Deviant paradigm) lasting approximately 5 min each, with each block comprising a fixed pseudorandom sequence of 615 tones. The order of the four MMN blocks was randomized. In order to reduce the influence of attention on the MMN measurements, participants were instructed to ignore auditory stimuli while performing simultaneously a computerized picture-word matching task presented on a video display that required a button press response on each trial (for task details, see Perez et al., 2014) [40].

### EEG data acquisition and preprocessing

EEG activity during the MMN paradigms was recorded in the ESZ participants before and after the AT or CG intervention. To confirm the presence of MMN amplitude reduction in the ESZ participants at baseline, we used EEG data from age-matched healthy control (HC) participants who had completed the same MMN paradigm in the context of other research studies. HC participants were recruited by advertisements and word-of-mouth. Those with a past or current DSM-IV Axis I disorder (based on a Structured Clinical Interview for DSM- IV) or a first-degree relative with a psychotic disorder, and or with a history of substance dependence or abuse within the past year, a history of a significant medical or neurological illness, or a history of head injury resulting in loss of consciousness were excluded.

EEG data were acquired using a high-impedance BioSemi Active Two recording system and a 64-channel electrode cap (Biosemi, Amsterdam, Netherlands). Continuous EEG data were digitized at a rate of 1024 Hz, referenced offline to averaged earlobe electrodes, high-pass filtered at 1 Hz, and segmented into 1000 ms epochs time-locked to the onsets of the various types of auditory stimuli (−500 to 500 ms). Vertical and horizontal electro-oculograms, recorded from electrodes above and below the left eye and at the outer canthi of both eyes, respectively, were used to correct EEG for eye movement and blink artifacts using a regression-based algorithm [82]. Additional processing details, including the sorted averaging method used to select trials for averaged event-related potentials (ERPs) match those described previously [83]. Following baseline correction by subtraction (−50 to 0 ms) of each EEG epoch, electrodes containing epochs with outlier values were replaced by interpolated values based on a routine implemented in a previously published automated EEG data cleaning algorithm [84]. Specifically, a spherical spline interpolation was applied to any channel and epoch determined to be a statistical outlier (|z| > 3) on one or more of four parameters, including variance (to detect additive noise), median gradient (to detect high-frequency activity), amplitude range (to detect pop-offs), and deviation of the mean amplitude from the common average (to detect electrical drift) [85]. Subsequently, epochs were rejected if they contained amplitudes greater than ±100 μV in any of the electrodes included in the analysis: F3, Fz, F4, C3, Cz, C4. In the next step, ERP averages for all stimulus types were determined using a sorted averaging method shown to reduce noise in the MMN waveform by averaging over the subset of trials that optimizes the estimated Signal to Noise Ratio (eSNR) [40, 83] for each subject. Briefly, single-epoch root mean squared (RMS) amplitude values for each trial are calculated and sorted in ascending order for each stimulus type. The subset of sorted trials selected for ERP averaging are associated with the largest eSNR, which is the ratio of the number of trials to the variance of the amplitude values across trials. The number of trials contributing to ERPs for each stimulus type did not differ between groups (all ps > .11). Following sorted averaging, ERPs for all stimulus types were low-pass filtered at 30 Hz, and then standard tone ERP waves were subtracted from Deviant tone ERP waves to derive difference waves. The MMNs were then identified in individual difference waves as the most negative peak between 90 and 290 ms for all Deviant types.

### Statistical correction for normal aging effects

Given the broad age range of our ESZ sample (minimum was 14, maximum was 36), additional steps were taken to control for normal brain development and aging effects. To this purpose, we used EEG data from 121 healthy control (HC) participants who had previously completed the same MMN paradigm. Normal aging effects on the MMN were modeled in the HC (age range 12–43 years) by regressing the MMN amplitudes on age separately for each Deviant type and electrode. Next, resulting regression models were used to derive predicted normal MMN amplitudes for each subject (ESZ and HC) based on their specific age. Finally, we divided the differences between observed and age-specific predicted MMN amplitudes by the standard error of regression (from the HC regression model), yielding age-corrected MMN *z-* scores. These z-scores represent, in standard units, the degree to which a subject’s (ESZ or HC) MMN amplitude deviates from the normal value expected for their age. Accordingly, more positive *z-*scores indicate smaller MMN amplitude relative to HC norms (i.e., a greater MMN deficit). For statistical comparisons of age-corrected MMN *z-*scores between the ESZ and HC groups, we only included the HC participants (*n* = 105) who were younger than the oldest ESZ patient (age = 36.17). Because z-scoring effectively sets the HC mean equal to zero, within-subjects effects were assessed in a separate model using raw MMN scores.

### Planned analyses

MMN amplitudes averaged over the six fronto-central electrodes (F3, Fz, F4, C3, Cz, C4) were used in the analyses. To examine the contribution of Single-deviant MMNs to the Double-Deviant MMN, we performed regression analyses in which we regressed DBL MMN onto DUR and FREQ MMN, for ESZ and HC participants separately.

Group differences between HC and ESZ in baseline MMN age-corrected *z-*scores were assessed using a 4-way repeated measures Analysis of Variance (ANOVA) with Group (SZ, HC) as the between-subjects factor and Deviant Type (DUR, FREQ, DBL), Fronto-Central Lead (Frontal, Central), and Lateral Lead (Left, Midline, Right) as within-subjects factors. Significant effects were parsed using follow-up F-tests of simple main effects. Greenhouse-Geisser non-sphericity correction was applied to within-subjects effects with more than two levels.

All subsequent analyses were exclusively conducted on ESZ participants who completed baseline and post-training EEG and cognitive assessments. Cognitive variables were screened and normally distributed after winsorising of outlying values [86]. Pearson correlations were used to assess the relationships between MMN *z-*scores and baseline MATRICS Global Cognition scores. Alpha was set to *p* = 0.05, two-tailed, for all statistical tests.

AT and CG groups were compared on change in Global Cognition and MMN age-corrected z-scores using repeated measures ANOVA. Effect sizes (Cohen’s *d*) were computed using the mean change scores of the AT and CG groups (post-treatment minus baseline) and the change score SDs of each subgroup.

Change in MMN *z-*scores as a function of Treatment Group was assessed using 5-way repeated measures ANOVA with Treatment Group (AT, CG) as the between-subjects factor and Deviant Type (DUR, FREQ, DBL), Fronto-Central Lead (Frontal, Central), and Lateral Lead (Left, Midline, Right), and Time (Baseline, Post-treatment) as within-subjects factors. Significant effects were parsed using *post-hoc* Tukey-Kramer tests. Greenhouse-Geisser non-sphericity correction was applied to within-subjects effects with more than two levels. Pearson correlations were used to examine associations between Global Cognition change scores and MMN *z-*change scores changes in AT participants.

To test whether baseline MMN *z-*scores predicted changes in Global Cognition scores, we performed a separate regression analysis for each MMN Deviant type in which we regressed the Global Cognition change score (post-treatment minus baseline) on Treatment Group (AT vs CG), baseline MMN *z-*score, and their interaction (baseline MMN *z-*score* Treatment Group). The interaction effect tests whether the slope of the relationship between Global Cognition change and MMN significantly differs between the groups.

The stability of the MMN signal was examined by calculating Intra-class correlations (ICCs) between baseline and post-training MMN raw and *z-*scores across Treatment groups for each MMN type separately. The stability of the trait aspects of MMN was operationalized as the average of baseline and post-training MMN *z-*scores. Twenty eight of the HC participants completed a second EEG session approximately 6 months (6.7 ± 1.8) after their baseline EEG session, allowing for ICC calculation in this HC sub-group as well.

Finally, we regressed Global Cognition change scores on Treatment Group (AT vs CG), trait-like MMN *z-*score, and their interaction. For all regression models, in case of statistically significant slope differences, we ran follow-up within-group Pearson correlations to estimate the strength of the correlation between the predictor and the dependent variable.

## Results

### Relationships among MMN measures in healthy controls and individuals with schizophrenia

In the HC group, baseline DUR and FREQ MMN *z-*scores were moderately correlated (*r* = .42, *p* < .001). DBL MMN similarly correlated with FREQ MMN (*r* = .63, *p* < .001) and DUR MMN (*r* = .49, *p* < .001). In the ESZ group, baseline DUR and FREQ MMN *z-*scores were moderately correlated (*r* = .52, *p* < .001). DBL MMN similarly correlated with FREQ MMN (*r* = .57, *p* < .001) and DUR MMN (*r* = .54, *p* < .001). When the DBL MMN was regressed on both frequency and duration MMN in a multiple regression model in the healthy controls, both accounted for independent aspects of the variance of the DBL MMN (DUR MMN R^2^ change = .24, *p* < .001, FREQ MMN R^2^ change = .21, *p* < .001), leaving 54% of the DBL MMN variance unaccounted for. A similar pattern of results was observed in the ESZ group (DUR MMN R^2^ change = .29, *p* < .001, FREQ MMN R^2^ change = .12, *p* = .002; DBL MMN variance unaccounted for =59%). Thus, while in ESZ the DBL MMN shared more variance with the DUR MMN than with the FREQ MMN, it contained information over and above that provided by the Single-Deviant frequency and duration MMNs.

### Group differences in baseline MMN between healthy controls and individuals with schizophrenia

**Fig. 1:**
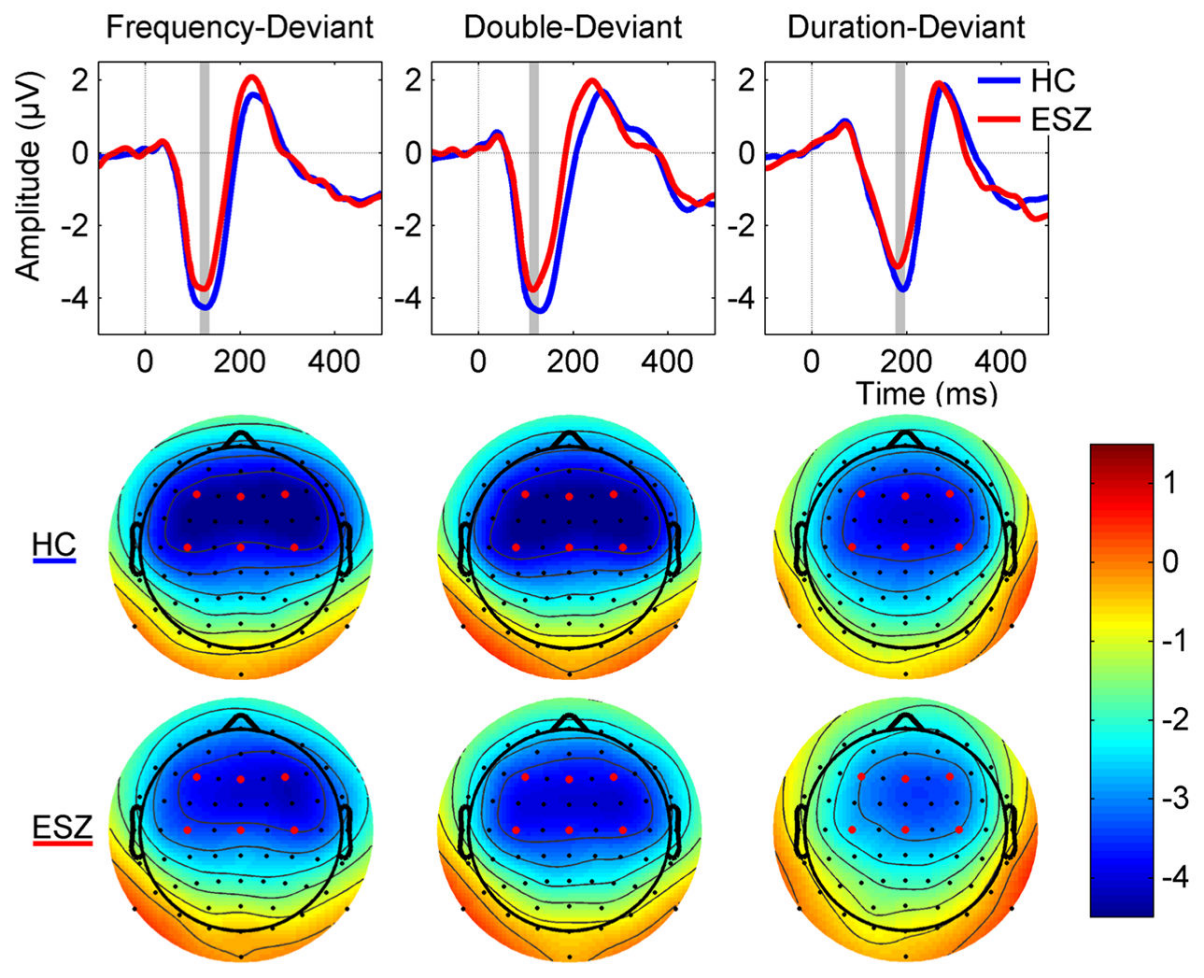
Mismatch Negativity (MMN) Amplitude For Each Group And Deviant Type. Ear-referenced ERP difference waveforms averaged across electrodes Fz, Fz, F4, C3, Cz, C4 for Duration, Double (duration + frequency), and Frequency MMN are given for each group (*top*). Healthy Controls (HC) are shown in *blue*, and individuals with Early Illness Schizophrenia (ESZ) in *red*. Scalp voltage topography maps of MMN amplitudes are shown for HC (*middle*) and ESZ (*bottom*) for each deviant type. MMN topography maps show the group means of MMN amplitudes around the grand average peak latency ±10 ms (indicated by gray bars in ERP difference waveform plots). *Red dots* on scalp topography maps indicate the 6 channels used in group comparisons and plotted in grand average waveforms. MMN is reduced in ESZ relative to HC across deviant types. Plotted data reflect group averages prior to any standardization based on the HC group

Results of the *Group x Deviant Type x Fronto-Central Lead x Lateral Lead* repeated measures ANOVA of MMN *z-*scores are presented in Table 1. In addition to a significant Group effect (*p* = .029) indicating an overall reduction of MMN amplitude (more positive *z-*score) in the ESZ relative to the HC, there was also a significant *Group x Fronto-Central Lead x Deviant Type* interaction (*p* = .005). This three-way interaction was parsed by examining the *Group x Fronto-Central Lead* effect for each deviant type. The *Group x Fronto-Central Lead* effect was significant for the DBL MMN (*p* = .006), but not for the DUR or FREQ MMN. Further parsing of this *Group x Fronto-Central Lead* interaction for DBL MMN indicated a significant Group effect at Frontal leads (*p* = .003), with ESZ showing MMN deficits compared to HC, and a non-significant Group effect at Central leads. We also parsed the three-way interaction by examining the *Group x Deviant Type* interaction separately at frontal and central leads, but in neither case was this effect significant. Taken together, these analyses indicate the presence of a DBL MMN amplitude deficit over frontal leads in the ESZ group compared to the HC group. Analysis of raw scores indicated a main effect of deviant type (F = 5.969, df = 2, *ε̂*=.921, *ε̂*-corrected *p* = .004) that was driven significantly larger DBL MMN amplitude than DUR (*p* = .002) but not FREQ (*p* = .469) MMN. Ear-referenced ERP difference waveforms and scalp voltage topography maps of MMN amplitudes for HC and ESZ are presented in Fig. 1.

### Baseline characteristics of the sample of individuals with schizophrenia

Within the ESZ group, greater impairment in baseline Global Cognition was associated with greater deficits in MMN amplitude (more positive *z-*scores), for both the DBL MMN (*r* = −.346, *p* = .009) and the FREQ MMN (*r* = −.329, *p* = .013), but not for DUR MMN (*r* = −.162, *p* = .233). Demographic data and mean MMN *z-*scores averaged across the six fronto-central leads for ESZ participants undergoing 40 h of AT vs. CG are presented in Table 2. There were no differences between treatment groups in demographics, baseline cognitive measures, symptom severity, and MMN age-corrected *z-*scores. Demographic data for HC are presented in Table 3.

### Effects of auditory training and computer games on cognitive outcomes

Mean Global Cognition pre- and post-treatment *z-*scores are presented in Table 4. Repeated measures ANOVA for Global Cognition revealed a marginally significant main effect of Time (F = 3.719, df = 1,54, *p* = .059) and a trend-level *Treatment Group x Time* interaction (F = 2.960, df = 1,54, *p* = .091) with small effect size (*d* = 0.25). Mean Global Cognition *z-*score changes and SDs were 0.25 ± 0.62 for the AT group and 0.01 ± 0.41 for CG group. Eighteen (67%) participants in the AT group and 8 (27%) participants in the CG group showed Global Cognition improvements beyond expected practice effects (0.2 SD) [21], a difference that was significant (*X* square = 9.54, *p* = .002).

### Effects of auditory training and computer games on MMN z-score amplitude

**Fig. 2:**
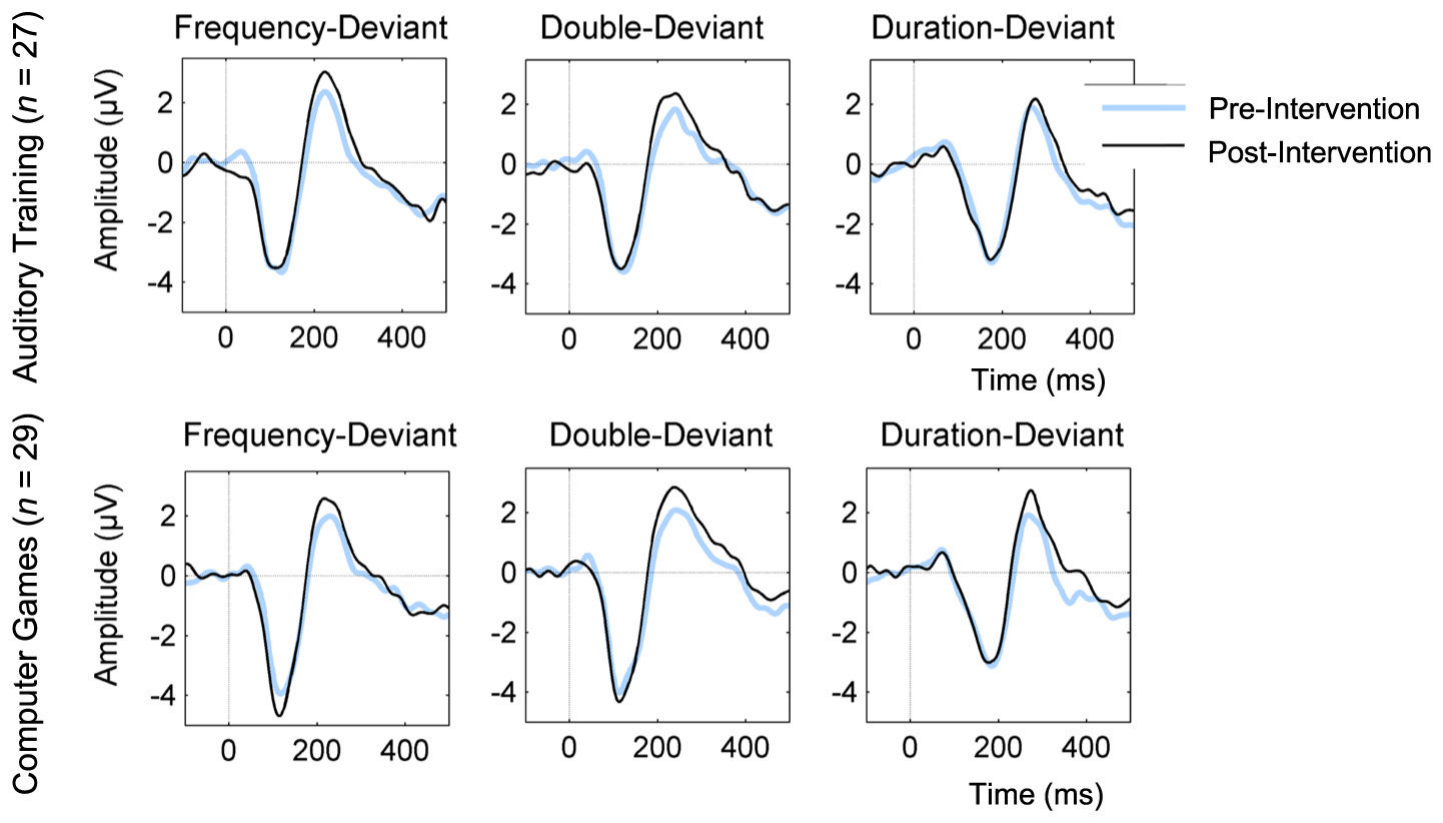
Mismatch Negativity (MMN) Amplitudes For Each Treatment Group And Deviant Type Before And After The Intervention. Ear-referenced ERP difference waveforms averaged across electrodes Fz, Fz, F4, C3, Cz, C4 for Duration, Double (duration + frequency), and Frequency MMN are given for Auditory Training (AT) participants (*top*) and Computer Games (CG) participants (*bottom*). Baseline MMN amplitudes are shown in *blue*, post-intervention MMN amplitude are shown in *black*

Mean MMN and pre- and post-treatment *z-*scores are presented in Table 4. In the ANOVA examining change in MMN *z-*scores between treatment groups (Table 4), we found a trend-level *Treatment Group x Deviant Type x Time* interaction (F = 2.889, df = 2, *ε̂* =.988, *ε̂*-corrected *p* = .061) and a significant *Treatment Group x Lateral Lead x Time* interaction (F = 4.609, df = 2; . *ε̂*=.891, *ε̂*-corrected *p* = .015). However, the *Treatment Group x Deviant Type x Lateral Lead x Time* interaction was not significant (F = 1.499, df = 4, *ε̂*=.698, *ε̂*-corrected *p* = .219). The *Treatment Group x Lateral Lead x Time* interaction were parsed in several ways. The *Treatment Group x Time* effect was examined for each each lateral scalp site separately, collapsing across fronto-central leads and deviant types. Here we found no significant *Treatment Group x Time* interactions for left, midline or right scalp sites. Additional parsing of the interaction effect is presented in Table 5. Ear-referenced ERP difference waveforms for pre- and post- treatment MMN amplitudes are presented in Fig. 2. Taken together, these findings seem to suggest that participants who undergo AT or CG do not show robust changes in MMN amplitude. Finally, we did not find significant associations between changes in Global Cognition and changes in MMN *z-*scores in AT and CG participants.

### Associations between baseline MMN and cognitive changes

Next, we tested a series of multiple regression models to evaluate whether baseline MMN predicted changes in Global Cognition, and whether such predictive relationships significantly differed between the two treatment groups. Models were run for each MMN deviant type. There were no significant slope differences between the groups for any of the MMN deviant types. After dropping the non-significant Group x MMN *z-*score interaction terms from the regression models, tests of the common slope predicting change in global cognition from baseline MMNs revealed significant effects for DBL MMN (*p* = .048) and DUR MMN (*p* = .013), and trend-level effects for FREQ MMN (*p* = .056), with greater MMN deficits predicting greater cognitive improvements across groups.

### Trait-like stability of MMN

Given the non-significant changes in MMN amplitude *z*-scores induced by the AT and CG interventions, we examined the extent to which individual differences in MMN amplitude *z-*scores among ESZ participants showed trait-like stability over the pre-post test interval (average duration = 70.15 days, all *p*s for between-groups differences were > .05), ignoring the small influence of treatment effects. This was done by simply calculating ICCs between baseline and post-training MMN raw and z-scores in the HC and ESZ samples. We found fair to good test-retest correlations for all MMN deviant types (ICCs are presented in Table 6, all *p*s < .001), suggesting trait-like stability and durable reliability of the MMN signal, despite the minor changes induced by the two treatments or by time in the HC sub-sample.

### Associations between trait-like MMN and cognitive changes

**Fig. 3:**
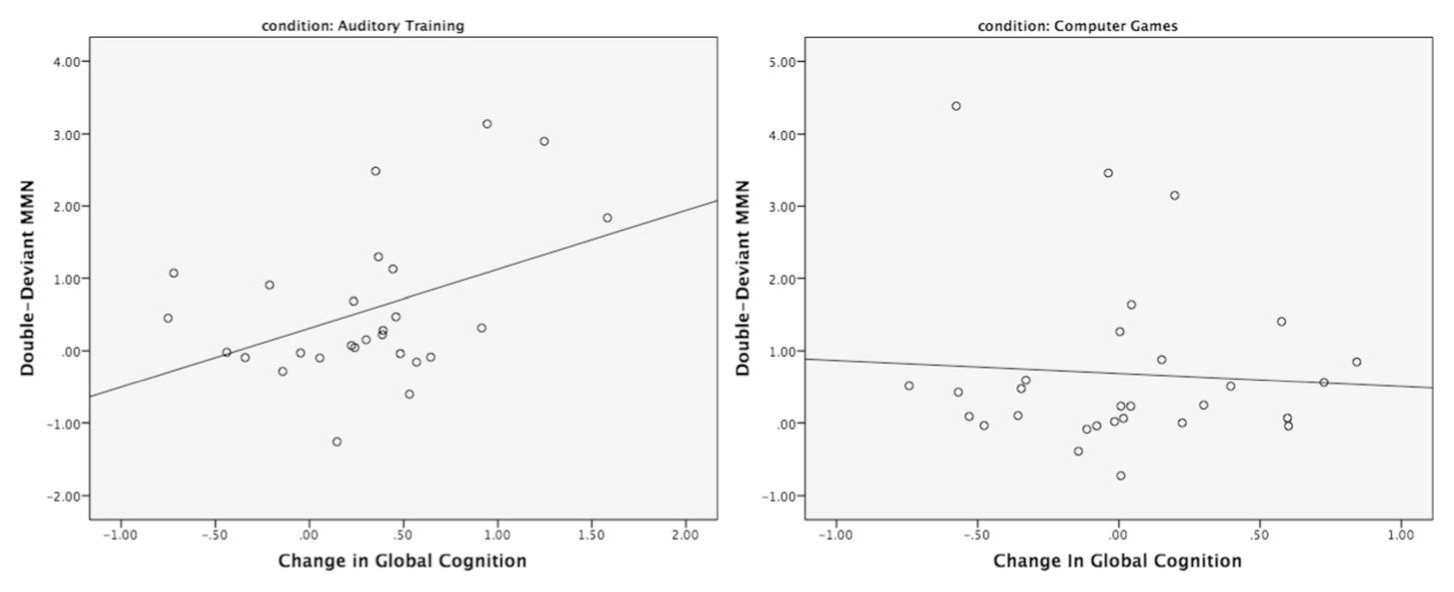
Scatter plots of the relationships between Change in Global Cognition and Trait-like Double-Deviant Mismatch Negativity (MMN) for Participants With Schizophrenia Who Received Computerized Auditory Training (*left*) and Participants Who Played Computer Games (*right*)

To best capture the trait aspects of MMN, and to enhance the reliability of the MMN measurements, we averaged the baseline and post-training MMN *z-*scores for each MMN deviant type and for the frontal and central leads separately. These trait-weighted MMN measures, reflecting more temporally stable aspects of MMN, were then entered into a series of regression models examining their relationship with pre-post changes in Global Cognition and possible differences in the slopes of these relationships between the two treatment groups. While there were no significant slope differences between the groups for any of the FREQ or DUR MMN deviant types, there was a significant Treatment Group x time-averaged MMN interaction (*p* = .05) for the DBL MMN (see Table 7). Follow-up Pearson correlations within each treatment group showed that greater trait-like deficits in DBL MMN amplitude (more positive *z*-scores) significantly predicted greater improvements in Global Cognition in the AT group (*r* = .442; *p* = .02), whereas this relationship in the CG group did not approach significance (*r* = −.05; *p* = .798). For scatter plots, see Fig. 3. This suggests that among participants receiving AT, those with greater trait-like Double-Deviant MMN deficits showed greater cognitive gains.

## Discussion

In this study, we directly compared the sensitivity of MMN elicited by three types of auditory deviance (Duration, Frequency, and Frequency + Duration Double-Deviant) in individuals with a schizophrenia-spectrum illness (ESZ) who were, on average, within 2 years of their illness onset, and healthy controls (HC). We found reduced MMN amplitude irrespective of deviant types in the ESZ group relative to the HC group, consistent with prior studies [38–40, 87]. In addition, we found baseline MMN deficits in the ESZ to correlate with worse general cognitive performance, replicating prior studies [48, 49]. These baseline correlations with general cognition were only evident for Frequency and Double-Deviant MMN, but not Duration MMN. In terms of the auditory training treatment effect on cognition, we found only a trend-level Treatment Group x Time interaction with small effect size (*d =* 0.25), although the larger study from which the current sub-sample was drawn did demonstrate significant benefits of auditory training relative to the computer game control condition, with a relatively large effect size for global cognition (*d =* 0.73) [17]. Importantly, despite the fact that the auditory training administered in the current study focused on auditory processing, we found no significant evidence of treatment-induced changes in MMN amplitude. We further examined whether baseline MMN amplitude could predict the cognitive changes induced by auditory training and computer games. We found that greater MMN deficits predicted greater cognitive improvements, with no significant slope differences between the treatment groups. Relatively high correlations were found between baseline and post-training MMN amplitudes for all deviant types when the two groups were combined, supporting the trait-like stability of the MMN over time despite the interventions administered. When the baseline and post-treatment MMN values were averaged together, emphasizing trait aspects of the MMN and enhancing measurement reliability, the correlation between Double-Deviant MMN deficits and cognitive gains in the auditory training group reached statistical significance. In particular, greater deficits in MMN elicited during the Double-Deviant paradigm predicted greater improvements in general cognitive performance after auditory training. This association was not present for Single-Deviant baseline MMNs, and not present in ESZ participants who underwent computer games.

Consistent with prior studies [38, 87], including ours [40, 83], we found that deviant type did not significantly matter in distinguishing ESZ from HC. This finding fails to support other evidence that Duration-Deviant MMN is more sensitive to schizophrenia than other types of MMN, particularly during the earlier phases of the illness [60, 88, 89].

Given the potential for heterogeneity among individuals with ESZ in terms of which type of MMN is most affected by their particular variant of the illness, we included a Double-Deviant paradigm to increase sensitivity to variations in MMN deficits between ESZ participants. While we did not find significantly greater sensitivity of Double-Deviant MMN to ESZ relative to the corresponding Single-Deviant MMNs, consistent with our prior studies in ESZ [83] and in individuals at clinical high risk for psychosis [40], Double-Deviant MMN correlated with the cognitive changes induced by auditory training, whereas the Single-Deviant MMNs did not. This finding may reflect the ability of Double-Deviant MMN to capture variation in both Frequency- and Duration-Deviant MMN among ESZ participants within a single measure. Of note, the correlations among the MMN measures in the ESZ group indicated that Double-Deviant MMN more strongly covaried with Frequency-Deviant MMN than with Duration-Deviant MMN. Additionally, the fact that only 41% of the variance of the baseline Double-Deviant MMN in ESZ and only 46% of the variance of the baseline Double-Deviant MMN in HC were explained by the contributions of Frequency-Deviant and Duration-Deviant MMN in regression models further indicates that Double-Deviant MMN reflects more than an additive index of the two Single-Deviant MMN measures. This is consistent with other studies in HC showing that Double-Deviant MMN captures neurophysiological processes associated with multi-feature auditory deviance detection that are not assessed by separate assessment of MMN to each deviance feature [70–72]. Interestingly, in our prior study of individuals at clinical high risk for psychosis [40], Double-Deviant MMN significantly predicted time to conversion to psychosis, even after controlling for Single-Deviant MMNs, further suggesting the potential utility of Double-Deviant MMN for improving sensitivity to some clinical and cognitive outcome measures.

We also found that greater deficits in MMN amplitude (more positive MMN *z*-scores), and not more intact MMN, were associated with larger cognitive gains induced by auditory training in ESZ. This supports the idea that those with greater auditory processing deficits stand to benefit the most from auditory training, but does not support the alternative hypothesis that greater MMN deficits may index impairments in the mechanisms of short-term synaptic plasticity on which auditory training depends, thereby identifying those who are less likely to show training-induced cognitive gains. Interestingly, this result contrasts with a recent report by Perez and colleagues [90], who showed that larger MMN amplitudes in individuals with schizophrenia at baseline and after 1 h of auditory training predicted greater auditory perceptual learning gains, although the study did not report on whether MMN amplitudes predicted the longer term cognitive testing gains examined in the current study.

Finally, we did not find that auditory training significantly improved MMN amplitude in ESZ participants, disconfirming the hypothesis that MMN could function as a proxy for the neurophysiologic changes in auditory processing produced by auditory training. While this finding was not predicted, it is noteworthy that in a recent study of individuals with schizophrenia, 1 h of the same auditory training program used in the current study induced both auditory perceptual learning gains and a significant reduction in MMN amplitude [90]. The authors hypothesized that the MMN amplitude decrement induced after 1 h of training may reflect transient short-term cortical perceptual reorganization indicative of the engagement of neuroplasticity-based mechanisms, and that with longer periods of training, MMN amplitude increases might emerge and accompany the expected improvements in cognition [90]. The authors further speculated that the changes induced by 1 h of auditory training might have affected the orientation of the cortical dipoles contributing to MMN, resulting in an apparent amplitude reduction at the scalp that might obscure an increase in MMN amplitude evident in source space [90]. In any case, it is clear that our results did not show any enhancement of MMN amplitude with auditory training. This is in line with findings from a recent study conducted by Kärgel and colleagues in schizophrenia that found that auditory training induced significant improvements in cognition but no changes on MMN amplitude or latency [79]. If the lack of MMN changes could have been ascribed to the small sample size, the narrow range of auditory stimuli, or the insufficient number of training sessions in the Kärgel et al. study, our rigorous testing of an intensive auditory training program in an adequately powered sample of ESZ participants seem to curtail those explanations.

We identified two possible explanations for the lack of enhancement of MMN amplitudes after auditory training. First, as magneto-encephalography and functional magnetic resonance imaging data have shown this treatment to improve brain function during early sensory and higher order cognitive processing in auditory and prefrontal cortices [11, 13, 91], it is plausible that MMN is simply not the appropriate index of neurophysiologic changes in the central auditory system. In the narrowest view of the MMN as simply a readout of the brain’s ability to automatically discriminate auditory stimuli, it might only be reasonable to expect MMN amplitude to increase post-training if the auditory training itself focuses on enhancing discrimination of the specific stimuli presented in the MMN paradigm. The auditory training used in the current study included exercises to enhance discrimination of frequency-modulated tones in the 761–2000 Hz range, as well as discrimination of more complex speech-related stimuli, whereas our MMN paradigms assessed the response to auditory deviance using a 633 Hz 50 msec standard tone and deviant tones that were either 1000 Hz 50 msec, 633 Hz 100 msec, or both. It is therefore possible that any plasticity generated in the auditory system by the training program is narrowly confined to the processing of the specific stimuli used, which did not exactly correspond to the stimuli used in our MMN paradigms. However, at least one study that did use identical auditory stimuli for the auditory training and the MMN paradigm still failed to show a change in MMN amplitude [79], suggesting that other factors contributing to MMN may diminish its sensitivity to changes induced by auditory training. In any case, the lack of significant associations between changes in general cognitive performance and changes in MMN amplitudes in participants undergoing auditory training supports the hypothesis that MMN is not sensitive to the neurophysiologic changes induced by auditory training. Second, the negative findings could be partially driven by a sampling bias. The ESZ participants who underwent MMN assessments are a sub-sample of participants recruited for a larger RCT that demonstrated stronger effects of auditory training on global cognition compared to those observed in this analysis [17]. To address this limitation, future studies should evaluate MMN along with other neurophysiological indexes in larger samples of ESZ individuals undergoing auditory training.

In this study, the trait-like contributions to the MMN signal were enhanced by averaging data collected before and after the intervention under investigation. This basic strategy for increasing the reliability of measurements (Spearman-Brown Prophecy formula, see Anastasi, 1982) [92] has rarely been applied to ERP assessments, but at least one prior study demonstrated the benefit of this strategy for enhancing sensitivity of ERP P300 measurements to clinical fluctuations over time in schizophrenia [88]. The ability to detect changes in MMN induced by interventions, or during illness progression in schizophrenia, would benefit from enhancement of the reliability of the MMN measurements by averaging over more than two measurement occasions at each assessment time point. Unfortunately, pragmatic and cost constraints, rather than scientific considerations, limit the feasibility of introducing this measurement strategy into most longitudinal study designs.

Our findings provide preliminary evidence for a future personalized medicine approach that uses reliably measured baseline MMN to identify which individuals with schizophrenia are mostly likely to benefit from auditory training. Moreover, more longitudinal research is needed using optimally reliable MMN measurements to assess whether MMN progressively changes over time during the transition from psychosis-risk states to full-blown psychosis, particularly in light of recent evidence that greater MMN amplitude deficits are present in individuals at clinical high risk (CHR) who subsequently converted to psychosis [51, 87] and in those at more imminent risk of developing psychosis [40, 93]. Similar longitudinal studies are needed to track whether and how MMN changes during the transition from early to chronic phases of schizophrenia.

## Conclusions

In conclusion, this study demonstrates that Double-Deviant MMN amplitude is a stable trait that is reduced in ESZ compared to HC. Moreover, the trait aspects of Double-Deviant MMN seem to predict response to auditory training over and above the Single-Deviant MMNs, with greater deficits (smaller MMN amplitudes) associated with larger cognitive gains. Given that the neuroanatomically distinct MMN generators associated with processing different dimensions of auditory deviance seem heterogeneously compromised in schizophrenia [94] and possibly in individuals at risk for psychosis, it seems likely that deviant-specific MMNs may evolve differently over the illness course and that no Single-Deviant MMN will be optimally sensitive to disease among individuals with schizophrenia [95]. Accordingly, implementing Double-Deviant MMN paradigms in future studies of schizophrenia or its prodrome, including studies of auditory training, has the potential to capture more of the inter-individual variability, and therefore account for greater proportions of the variance in clinical and cognitive function evident in the disorder.

## Figures and Tables

**Table 1:** Analysis of Variance (ANOVA) of Mismatch Negativity (MMN) *z-*scores. More positive *z-*scores indicate smaller MMN amplitude relative to HC norms (i.e., a greater MMN deficit)

Effect	DF	F	*p-v*alue	Follow-up tests^a^
Group	1159	6.138	**0.029**	ESZ > HC
Deviant Type (DUR, FREQ, DBL)	1.929	0.431	0.643	
Fronto-Central Lead (Frontal, Central)	1	1.5306	0.218	
Lateral Lead (Left, Midline, Right)	1.760	3.217	**0.048**	
Group*Deviant Type	1.929	0.472	0.617	
Group*Fronto-Central Lead	1	1.043	0.309	
Group*Lateral Lead	1.760	1.992	0.144	
Group*Lateral Lead*Deviant Type	3.377	0.604	0.632	
Group*Fronto-Central Lead*Deviant Type	1.992	5.326	**0.005**	
Group*Fronto-Central Lead for DUR	1	0.338	0.562	
Group*Fronto-Central Lead for FREQ	1	0.041	0.839	
Group*Fronto-Central Lead for DBL	1	7.625	**0.006**	
Fronto-Central Lead effect in ESZ	55	4.336	**<0.001**	Frontal > Central
Group Effect at Frontal Leads	1156	8.828	**0.003**	ESZ > HC
Group Effect at Central Leads	1156	2.608	0.108	
Fronto-Central Lead *Deviant Type in ESZ	1.832	10.659	**<0.001**	
Deviant Type effect at Frontal Leads	1.964	2.571	0.082	DBL > DUR
Deviant Type effect at Central Leads	1.975	0.393	0.673	
Fronto-Central Lead effect for DUR	55	−0.906	0.369	
Fronto-Central Lead effect for FREQ	55	−0.169	0.866	
Fronto-Central Lead effect for DBL	55	4.336	**<0.001**	Frontal > Central
Group*Deviant Type at Frontal Leads	1.922	1.449	0.237	
Group*Deviant Type at Central Leads	1.938	0.325	0.716	
Group*Fronto-Central Lead*Lateral Lead	1.776	0.787	0.443	

*HC* healthy controls, *ESZ* early schizophrenia participants

*FREQ* Frequency Deviant, *DUR* Duration Deviant, *DBL* Frequency + Duration Double-Deviant

Statistically significant *p-*values are formatted in bold

a Between deviant-type comparisons are based on Helmert contrasts

**Table 2:** Baseline Demographics of Participants with Schizophrenia Who Received Computerized Auditory Training and Participants Who Played Computer Games

	Auditory Training (*n* = 27)		Computer Games (*n* = 29)				
	Mean	SD	Mean	SD	t	DF	*p-v*alue
Age, years	23.75	4.16	21.98	3.60	1.71	54.00	0.09
Education, years	13.00	1.78	12.72	2.95	0.41	50.00	0.69
FSIQ IQ^a^	104.44	13.68	101.90	14.58	0.67	54.00	0.50
Duration of illness, years	1.50	1.23	1.85	1.41	−0.96	51.00	0.34
Hours of training	37.33	6.30	39.62	1.37	−1.91	54.00	0.09
PANSS Symptom Scores							
Positive (mean of 7 items)	12.56	4.05	14.04	4.90	−1.18	50.00	0.24
Negative (mean of 7 items)	16.40	6.18	17.19	6.62	−0.44	50.00	0.66
General (mean of 16 items)	32.36	6.89	34.85	9.65	−1.06	50.00	0.29
MATRICS Global Cognition z-score	−1.12	0.93	−0.96	0.85	−0.66	54.00	0.52
DBL MMN *z-*score	0.35	0.99	0.44	0.88	−0.36	54.00	0.72
DUR MMN *z-*score	0.34	0.92	0.19	1.02	0.57	54.00	0.57
FREQ MMN *z-*score	0.32	1.00	0.43	0.99	−0.42	54.00	0.68
	n	%	n	%		χ2	*p*-value
Gender						0.319	0.572
Female	10	0.3	7	0.24			
Male	17	0.63	22	0.76			
Handedness^b^						0.248	0.884
Right	25	0.93	26	0.90			
Left	1	0.04	2	0.07			
Ambidextrous	1	0.04	1	0.03			
Antipsychotic type						0.305	0.662
Atypical alone	25	0.93	26	0.90			
Typical alone	0		1	0.03			
Both	0		0				
None	2	0.07	2	0.07			
Psychiatric comorbidities^c^							
Anxiety Disorder	0		0				
Depressive Disorder	3	.11	2	0.07			.661
Substance Abuse	3	.11	1	0.03			.341
Substance Dependence	3	.11	2	0.07			.661

Gender and handedness were analyzed with Pearson chi-square tests. The remaining demographic variables were analyzed with independent samples t-tests

*MMN* Mismatch Negativity

*FREQ* Frequency Deviant, DUR = Duration Deviant, DBL = Frequency + Duration Double-Deviant

*PANSS* Positive and Negative Syndrome Scale

*MATRICS* Measurement and Treatment Research to Improve Cognition in Schizophrenia

a Wechsler Abbreviated Scale of Intelligence (WASI)

b The Crovitz-Zener (1962) questionnaire was used to measure handedness

c Fisher’s Exact Tests were used to compare psychiatric comorbidity rates

**Table 3:** Baseline Demographics of Healthy Controls

	HC (baseline, *n* = 105)		HC (time 2, *n* = 28)	
	Mean	SD	Mean	SD
Age, years	23.01	6.35	23.11	5.52
Education, years	13.12	3.02	13.92	2.59
	n	%	n	%
Gender				
Female	44.00	0.42	14.00	0.50
Male	61.00	0.58	14.00	0.50
Handedness^a^				
Right	96.00	0.91	27.00	0.96
Left	8.00	0.08	1.00	0.04
Ambidextrous	1.00	0.01	0.00	0.00

Gender and handedness were analyzed with Pearson chi-square tests

The remaining demographic variables were analyzed with independent samples t-tests

a The Crovitz-Zener (1962) questionnaire was used to measure handedness

**Table 4:** Global Cognition and Mismatch Negativity (MMN) Amplitude *z-scores* for Participants With Schizophrenia Who Received Computerized Auditory Training and Participants Who Played Computer Games

	Auditory Training (*n* = 27)				Computer Games (*n* = 29)						
		
	Baseline		Post		Baseline		Post				
					
Outcome Measure^b^	Mean	SD	Mean	SD	Mean	SD	Mean	SD	F^a^	*p-v*alue	Effect Size
Global Cognition	−1.12	0.93	−0.87	0.98	−0.96	0.85	−0.95	0.94	2.96	0.09	0.25
DBL MMN^c^	0.35	0.99	0.46	0.81	0.44	0.88	0.38	0.89	0.60	0.44	0.18
DUR MMN^c^	0.34	0.92	0.28	0.73	0.19	1.02	0.34	0.85	0.89	0.35	−0.23
FREQ MMN^c^	0.32	1.00	0.45	1.10	0.43	0.99	0.10	1.02	4.83	**0.03**	0.46

*FREQ* Frequency Deviant, *DUR* Duration Deviant, *DBL* Frequency + Duration Double-Deviant

*MATRICS* Measurement and Treatment Research to Improve Cognition in Schizophrenia

a Repeated measures ANOVA, Treatment Group x Time interaction

b Cognitive measures were transformed to *z-*scores using normative data of healthy samples. Global Cognition is the average *z-*score across all measures from the MATRICS

c Group mean and standard deviation (SD) mismatch negativity (MMN) age-corrected z-scores for each deviant type averaged over the six fronto-central leads (F3, Fz, F4, C3, Cz, C4)

Statistically significant *p*-values are formatted in bold

**Table 5:** ANOVA of Treatment (AT vs CG) Effects on Mismatch Negativity (MMN) for Participants with Schizophrenia

Effect	DF	F	*p-v*alue
Time	1	0.01	0.919
Deviant Type (DUR, FREQ, DBL)	1.942	0.789	0.454
Fronto-Central Lead (frontal, central)	1	2.004	0.163
Lateral Lead (left, midline, right)	1.665	4.45	**0.020**
Time * Deviant Type * Treatment Group	1.977	2.889	0.061
Time * Lateral Lead * Treatment Group	1.782	4.609	**0.015**
Time*Treatment Group for left	1	0.143	0.707
Time*Treatment Group for midline	1	1.343	0.252
Time*Treatment Group for right	1	0.414	0.523
Time * Fronto-Central Lead * Treatment Group	1	0.277	.601
Time * Deviant Type * Fronto-Central Lead * Treatment Group	1.938	.066	.932
Time * Deviant Type * Lateral Lead * Treatment Group	2.792	1.499	.219
Time * Fronto-Central Lead * Lateral Lead * Treatment Group	1.993	.169	.844

*AT* Auditory Training, *CG* Computer Games

*FREQ* Frequency Deviant, *DUR* Duration Deviant, *DBL* Frequency + Duration Double-Deviant

Statistically significant *p-*values are formatted in bold

**Table 6:** Intraclass correlations (ICCs) of 6 site average MMN amplitude and z-scores in early illness schizophrenia patient (ESZ) and healthy control (HC) test re-test data

MMN type	HC (raw μV)	ESZ (raw μV)	HC (z-score)	ESZ (z-score)
Double-Deviant	0.7398	0.5715	0.7247	0.5902
Duration-Deviant	0.7181	0.5868	0.7105	0.5889
Frequency-Deviant	0.6918	0.6832	0.6597	0.6765

**Table 7:** Regression of Change in Global Cognition on Trait-like Double-Deviant Mismatch Negativity (MMN), Treatment Group, and their Interaction

	Beta	t	Sig. t	R^2^	R^2^ Change	F for R^2^ Change	*p-v*alue
Step 1				.125	.125	3.725	.031
Treatment Group (AT vs CG)	.322	2.480	.016				
Trait-like DBL MMN	.165	1.269	.210				
Step 2				.189	.064	4.009	.050
Treatment Group (AT vs CG)	.177	1.218	.229	.			
Trait-like DBL MMN	−.051	−.307	.760				
Treatment Group* Trait-like DBL MMN	.358	2.002	.051				

## Abbreviations

AT: Auditory Training
CG: Computer Games
DBL: Frequency + Duration Double-Deviant
DUR: Duration Deviant
EEG: Electroencephalogram
ERP: Event-related potential
ESZ: Early in the course of a schizophrenia-spectrum illness
FREQ: Frequency Deviant
HC: Healthy Controls
MATRICS: Measurement and Treatment Research to Improve Cognition in Schizophrenia
MMN: Mismatch Negativity
NMDA: *N*-methyl-D-aspartate
PANSS: Positive and Negative Syndrome Scale
RCT: Randomized clinical trial
